# Sex differences in viral entry protein expression, host responses to SARS-CoV-2, and in vitro responses to sex steroid hormone treatment in COVID-19

**DOI:** 10.21203/rs.3.rs-100914/v1

**Published:** 2020-11-04

**Authors:** Mengying Sun, Rama Shankar, Meehyun Ko, Christopher Daniel Chang, Shan-Ju Yeh, Shilong Li, Ke Liu, Guoli Zhou, Jing Xing, Austin VanVelsen, Tyler VanVelsen, Shreya Paithankar, Benjamin Y. Feng, Krista Young, Michael Strug, Lauren Turco, Zichen Wang, Eric Schadt, Rong Chen, Xiaohong Li, Tomiko Oskotsky, Marina Sirota, Benjamin S. Glicksberg, Girish N. Nadkarni, Adam J. Moeser, Li Li, Seungtaek Kim, Jiayu Zhou, Bin Chen

**Affiliations:** Michigan State University; Michigan State University; Institut Pasteur Korea; Michigan State University; Michigan State University; Sema4; Michigan State University; Michigan State University; Michigan State University; Michigan State University; Michigan State University; Michigan State University; Michigan State University; Michigan State University; Michigan State University; Spectrum Health; Sema4; Sema4; Sema4; Van Andel Research Institute; University of California; University of California; Icahn School of Medicine at Mount Sinai; Icahn School of Medicine at Mount Sinai; Michigan State University; Sema4; Institut Pasteur Korea; Michigan State University; Michigan State University

**Keywords:** sex difference, COVID-19

## Abstract

Epidemiological studies suggest that men exhibit a higher mortality rate to COVID-19 than women, yet the underlying biology is largely unknown. Here, we seek to delineate sex differences in the expression of entry genes *ACE2* and *TMPRSS2*, host responses to SARS-CoV-2, and in vitro responses to sex steroid hormone treatment. Using over 220,000 human gene expression profiles covering a wide range of age, tissues, and diseases, we found that male samples show higher expression levels of *ACE2* and *TMPRSS2*, especially in the older group (>60 years) and in the kidney. Analysis of 6,031 COVID-19 patients at Mount Sinai Health System revealed that men have significantly higher creatinine levels, an indicator of impaired kidney function. Further analysis of 782 COVID-19 patient gene expression profiles taken from upper airway and blood suggested men and women present profound expression differences in responses to SARS-CoV-2. Computational deconvolution analysis of these profiles revealed male COVID-19 patients have enriched kidney-specific mesangial cells in blood compared to healthy patients. Finally, we observed selective estrogen receptor modulators, but not other hormone drugs (agonists/antagonists of estrogen, androgen, and progesterone), could reduce SARS-CoV-2 infection in vitro.

## Main Text

A growing body of epidemiological evidence suggests that men exhibit a higher mortality rate to COVID-19 than women ^1–3^, yet the underlying biology remains largely unknown. Hypotheses pertaining to the expression of viral entry protein, hormone levels, and immune systems are actively explored ^4,5^ and sex steroid hormone drugs are being investigated in clinical trials (Estradiol: NCT04359329, Progesterone: NCT04365127, Degarelix: NCT04397718). In a recent study, sex differences in immune responses in COVID-19 were examined by Takahashi et al., where blood samples from female patients were found to present more robust T-cell activation than male patients during SARS-CoV-2 infection ^6^. Comparing samples from nasopharyngeal swabs, Lieberman et al. observed that male patients had reduced B cell-specific and NK cell-specific transcripts and an increase in inhibition of nuclear factor kappa-B signaling^7^.

SARS-CoV-2 engages the receptor ACE2 (angiotensin-converting enzyme 2) for entry into the target cell through its spike protein ^8^. Its internalization requires priming of the spike protein by the cellular protease TMPRSS2 (transmembrane protease, serine 2) in the host cell ^9^, thus co-expression of ACE2 and TMPRSS2 on the target cell surface is required for virus entry. The high mortality in patients with COVID-19 may be partially driven by the strong affinity of the virus to ACE2 and the facilitation from TMPRSS2. A few studies ^10–12^ analyzed gene expression of *ACE2* and/or *TMPRSS2* between sexes using bulk or single-cell RNA-Seq samples primarily profiled from healthy individuals. However, many of the hospitalized COVID-19 patients have an underlying illness which increases mortality risks, and are in older age range (>60 years) ^13^. Furthermore, the number of patients in these studies ^10–12^ was relatively small.

In this study, we address the question of sex differences in response to SARS-CoV-2 in three ways. First, in order to quantify expression differences between sexes, we leverage public gene expression profiles covering a wide range of age, tissues, and disease conditions, and later utilize Electronic Medical Records (EMR) to validate findings. We also harness the emerging COVID-19 patient gene expression profiles to characterize cellular response differences between sexes in the upper airway and blood. Lastly, we investigated *in vitro* antiviral activity of sex steroid hormones in two cell lines infected by SARS-CoV-2.

### Expression of ACE2 and TMPRSS2 in a diverse and comprehensive set of human samples

We first compiled three large independent expression datasets consisting of 220,835 samples from diverse tissue types and patient populations (healthy and disease conditions) and completed their meta-information, including sex, age group (younger: 0–19, middle: 20–59, and older: >60), and tissue of origin (14 main tissues), through machine learning and manual annotation (Figure S1). To minimize batch effects, all the samples in each dataset were profiled under the same platform and processed using the same pipeline. The first dataset was compiled from the Treehouse project (T), where 17,654 RNA-Seq samples primarily from consortium projects including TCGA, GTEx, and TARGET were processed through the Toil pipeline ^14^. The second dataset was downloaded from the ARCHS4 (A) project, where 60,936 human RNA-Seq samples profiled under the Illumina HiSeq 2000 platform were aligned using Kallisto ^15^. The last dataset was collected from the GEO (G), where 145,947 samples profiled under the Affymetrix GPL570 platform were processed using Robust Multi-array Average (RMA). The sex, age group, and tissue of origin were obtained from the original resources (Methods); however, a substantial number of samples had missing metadata, especially in sets A and G, where only 1,407 and 4,392 samples have all sex, age, and tissue information, respectively. Leveraging their expression profiles, we built machine learning models (deep multi-task neural network and XGBoost) that completed metadata for the majority of samples with high confidence (Table S1). All of these predictions were further manually inspected based on unstructured sample metainformation available in the source files when possible.

Since both the proportion of samples with high expression of entry proteins and the absolute expression value of these proteins within individual samples are important to understand sex differences, we analyzed both categorical and continuous expression data. We first merged all three datasets into one single matrix (referred to as the Merged dataset) consisting of 220,835 samples, after categorizing them into high (e.g., top 10% within each dataset) and normal expression groups (i.e., *ACE2* high *vs*. *ACE2* normal, *TMPRSS2* high *vs. TMPRSS2* normal, and *ACE2*&*TMPRSS2* high *vs*. *ACE2*&*TMPRSS2* normal) in individual datasets. The Merged dataset, including diseased samples, healthy samples, and those samples with the treatment of perturbgenes samples, might be one of the best resources to investigate expression of entry proteins thus far. Similarly, we compiled a single matrix consisting of 8,066 healthy samples from T (referred to as the Healthy dataset). Logistic regressions were applied to predict the high expression group using age, tissue, and sex as features (by default, 95% confidence interval (CI), female as a reference). In addition to analyzing categorical expression data using the Merged dataset, we compared absolute expression between sex groups for each dataset separately.

We did not observe any significant difference in the proportion of highly expressed *ACE2*, *TMPRSS2,* or *ACE2*&*TMPRSS2* samples between women and men in the Heathy dataset after adjusting for age and tissue (Table1). However, the proportion of highly expressed samples in men is larger than in women in the Merged dataset (*ACE2*: OR 1.25 [1.19–1.30], P < 0.001; *TMPRSS2*: OR 1.28 [1.23–1.34], P < 0.001; *ACE2*&*TMPRSS2*: OR 1.16 [1.09–1.24], P < 0.001) (Table 1). In the older group, proportion difference was also observed in the Merged dataset (*ACE2*: OR 1.15 [1.07–1.23], P < 0.001; *TMPRSS2*: OR 1.32 [1.24–1.42], P < 0.001; *ACE2*&*TMPRSS2*: OR 1.12 [1.03–1.22], P=0.009), but not in the Healthy dataset (Table 1). Neither *ACE2* nor *TMPRSS2* is highly expressed in the majority of samples, while both are indeed highly expressed in a considerable number of samples in both men and women suggested by the long tails in both G and A, but not in T (normal) (Figure 1A). Compared to the younger group (0–19), the older group (>60) has a larger difference of *ACE2* expression between males and females (G: M/F 1.11 [1.1–1.12], P < 0.001 in older vs. M/F 0.99 [0.98–0.99], P < 0.001 in younger) (Figure 1A) as well as *TMPRSS2* expression (G: M/F 1.04 [1.03–1.04], P < 0.001 in older vs. M/F 1.0 [0.99–1.0], P=0.58 in younger) (Figure S2A). Further analysis of additional disease samples with the highest expression of *ACE2* revealed that Crohn’s disease, ulcerative colitis, Barrett’s esophagus, trachoma, and ichthyosis have overall higher *ACE2* expression in disease samples compared to control (Student’s t-test, P < 0.05, Figure S3). The difference of *ACE2* expression between sexes in these disease samples was not observed, likely due to the small sample size for each disease. In summary, although expression difference of entry proteins between sexes was not observed in the Healthy dataset, higher *ACE2* expression was found in men, especially in older men, in the Merged dataset.

Next we investigated whether there are expression differences in individual tissues. Perhaps because of the wide coverage of samples in A and G, expression of *ACE2* has a larger variation in both datasets than in the Healthy dataset T, especially in the kidney, small intestine, heart, liver, and colon (Figure 1B), while a large variation exists in the expression of *TMPRSS2* in the kidney, small intestine, liver, colon, lung, pancreas, and prostate (Figure S2B). *ACE2* is not differentially expressed between sexes in the lung (OR: 0.9 [0.78–1.04], P > 0.001), and women have even lower *TMPRSS2* expression in the lung in the Merged dataset [OR: 0.71(0.64–0.78), P < 0.001] (Table S2). Notably, the kidney is the only tissue showing a remarkable difference in *ACE2* expression between sexes in both A and G (Figure 1B). After adjusting for age and data source, the OR is 1.45 [1.26–1.67] (P < 0.001) (Table S2). The kidney is also the only tissue showing a significant difference in *TMPRSS2* expression between sexes in both A and G (Figure S2B). We were able to further map 28% of those samples with high *ACE2* expression to their diseases based on sample metainformation. The top mapped diseases are clear cell/renal cell carcinoma (60.8%), renal interstitial fibrosis (9.1%), acute kidney injury (7.5%), glomerulosclerosis (6.7%), nephritis (4.3%), and nephropathy (2.6%). In addition, as steroid hormone receptors regulate the renin-angiotensin-aldosterone-system, where ACE2 is an essential component ^16^, we examined the expression relationship between ACE2 and steroid hormone receptors. *ACE2* expression has a higher correlation with *AR* expression (Androgen Receptor, Spearman Rho: 0.72, P < 0.001) than with *ESR1* expression (Estrogen Receptor 1, Rho: 0.19, P < 0.001), *ESR2* expression (Estrogen Receptor 2, Rho: −0.12, P < 0.001), and *PGR* expression (Progesterone Receptor, Rho: 0.26, P < 0.001) in the kidney (Figure S4A). The genes regulated by AR also highly overlap with the genes positively co-expressed with *ACE2* in the healthy adrenal gland (P = 3.08E-5, Figure S4B), suggesting that *ACE2* expression might be associated with androgen receptor activity in the kidney.

In order to find clinical evidence of these findings, we analyzed 6,031 COVID-19 patients (4,621 inpatients and 1,410 outpatients with available labs) for serum creatinine levels measured in five member hospitals at Mount Sinai Health System up to May 10, 2020. We observed that men have significantly higher serum creatinine levels than women after normalizing to sex-specific reference ranges and adjusting for age and race (Inpatients OR: 1.89 [1.66–2.15], P < 0.001; Outpatients OR 2.12 [1.68–2.66], P < 0.001) (Extended Data), indicating COVID-19 male patients are most likely to have kidney dysfunction than female patients. Whereas, both expression and clinical data analysis suggest that sex difference in the kidney is not specific to the older group (Table S2 and Extended Data). Recent studies reported that acute kidney injury is common in patients hospitalized with COVID-19 and is associated with increased mortality ^17,18^. Together, the expression difference of entry proteins in kidney between sexes might be a factor contributing to sex differences in COVID-19 susceptibility.

### Sex stratified analysis of host responses to SARS-CoV-2

We searched GEO and SRA to obtain COVID-19 patient RNA-Seq samples and reprocessed raw sequence data when possible. We compiled four datasets with gender information (one from upper airway nasopharyngeal, one from upper airway naso/oro-pharyngeal, one from blood PBMC, and one from blood leukocytes), totaling 782 samples (Table S4). In each dataset, the ratio of the number of samples between sexes is close to 1. For each large upper airway dataset, we stratified samples into an older age group (>60 years) and a middle age group (20–60 years). We enumerated all the possible comparisons for each dataset (i.e., female control vs. female patient, male control vs. male patient, female control vs. male control, and female patient vs. male patient), with each comparison using the same thresholds to select differentially expressed genes. In comparing female and male samples either in the control group or in the COVID-19 group, only a few sex-specific genes were dysregulated between women and men. However, expression of a vast number of genes was significantly changed (p < 0.001, absolute fold change > 2) between healthy patients and COVID-19 patients in either men or women (e.g., 4269 DE in female CT vs. SARS2 and 911 DE in male CT vs. SARS2 in middle age group; 627 DE in female CT vs. SARS2 and 29 DE in male CT vs. SARS2 in older age group in GSE152075) (Figure 2 A-F). Interestingly, such changes seem unique to each sex with only a small portion of DE genes shared by both sexes. The two datasets from blood show the largest number of shared DE genes (35.7% and 30.8%, Figure 2E, 2F), while the dataset from older male upper airway has the lowest number of shared DE genes (0.1%, Figure 2B). The lower number is likely because fewer genes were differentially expressed in older male upper airways after SARS-CoV-2 infection Female patients presented very distinct gene expression changes in all datasets, especially in the younger group (Figure 2A, 2C). Pathway enrichment analysis of these distinct DE genes confirmed the immune response differences (cytokinin mediated signaling, cellular response to interferon-gamma and interferon 1) as previously reported ^6,7^; however, a few other non-immune related pathways were enriched in female patients, including down-regulation of mitochondrial respiratory responses and regulation of cholesterol biosynthesis (Figure 2 G-I). The younger male group presented the downregulation of various immune responses such as humoral immune response, acute inflammatory response, and Fc-gamma signaling pathways (Figure 2G), but no enriched pathways were observed in older male COVID-19 patients. Together with the higher susceptibility in older men, the analysis suggests that men and women have distinct host cellular responses in addition to immune responses. Importantly, out dataset suggests that weak host responses in the upper airway could be one indicator of susceptibility.

We further inferred the enrichment of 64 cell types in COVID leucocytes samples using Xcell ^19^ and compared cell type enrichment. In both men and women, CD8+ T-cells and memory CD8 T Cell were suppressed in COVID ICU patients (Figure S5). NK cells were suppressed in male ICU patients while neutrophils were elevated in female ICU patients. One striking difference between men and women came from the enrichment of mesangial cells, a kidney-specific cell type (control vs. COVID: male p-value of 1E-7 and female p-value of 1E-1, Student’s t-test). Logistic regression analysis of enrichment of mesangial cells with disease severity (non-ICU vs. ICU) indicated patients with higher enrichment of mesangial cells are more likely admitted into ICU [OR: 3.2(1.6–8.3), P < 0.001] (adjusted for age, Supplementary Figure S5). Together with the higher expression of *ACE2* and higher creatine levels in men, this analysis implies that impaired kidney function could be one source of sex differences.

### Responses to sex steroid hormone treatment

The difference in sex hormone levels between sexes might contribute to disease susceptibility; however, to establish the connection requires the development of robust SARS-CoV-2 animal models or the launch of clinical trials, either of which could not be accomplished soon. Therefore, we sought to understand how infected cells respond to the treatment of hormones *in vitro*, including estrogens, progesterone, and androgens. We first evaluated the anti-SARS-CoV-2 activity of estradiol (estrogen receptor agonist), fulvestrant (estrogen receptor antagonist), danazol (androgen receptor agonist), bicalutamide (androgen receptor antagonist), and hydroxyprogesterone caproate (OHPC, progesterone receptor agonist) in Vero and Calu-3 cells (Table 2, Figure S6). Among these drugs, only OHPC was effective in cells challenged with SARS-CoV-2 (IC50 13 µM in Vero and 6.4 µM in Calu-3). A previous study validated progesterone *in vitro* and proposed it might act through targeting sigma receptors, the inhibitors of which displayed antiviral activity *in vitro* ^20^. Thus, we evaluated an additional progesterone receptor agonist desogestrel and did not observe the efficacy (IC_50_ > 50 µM). Similarly, in an independent screening effort from the NCATS OpenData Portal project, three progesterone receptor agonists (desogestrel, chlormadinone acetate, and danazol) showed weak anti-cytopathic effect activity (20 µM) ^21^. This suggested that the potential protective effect of progesterone might come from its off-target effect on sigma receptors. We further surveyed the activity of 62 steroid and non-steroid hormones drugs through literature search and querying of large-scale screening databases including NCATS OpenData Portal, and confirmed that ER agonists, ER antagonists, AR agonists, AR antagonists, and PR agonists generally did not present *in vitro* anti-SARS-CoV-2 activity, except diethylstilbestrol, a non-steroid ER agonist (with IC_50_ of 4.5 µM) (Extended Data). However, six out of eight selective estrogen receptor modulators (SERM) showed considerable activity (IC50: 3.4–12 µM). SERM also presented anti-EBOV activity in previous screening efforts, and their activity appeared to be an off-target effect ^22,23^. Together, the role of hormones in antiviral activity is still inconclusive; however, our data are hopeful to incite deeper investigation of its effect *in vivo* or in the clinic.

## Discussion

COVID-19-related death is mainly associated with being male, older age, and comorbidities ^26^. The virus first enters the nose and throat, and then travels down to attack lung, which likely causes substantial respiratory pathology including acute respiratory distress syndrome. Its reach can extend to many other organs like blood vessels, liver, kidney, heart, and brain ^27^. ACE2 and its partner proteins are the key facilitators of virus entry into different organs. Therefore, their expression levels could be associated with disease severity and further mortality, and their expression differences between sexes could partially explain the higher mortality in men. Previous efforts did not detect the difference ^10,11^, likely because only healthy tissues were examined. In our Healthy dataset, we did not observe the difference either. Therefore, a comprehensive dataset covering a wide range of tissue samples including diseased samples is necessary to solve the puzzle. In order to draw robust conclusions, we utilized two large independent datasets based on distinct technologies: microarray and RNA-Seq. In addition, we examined the differences according to the percentage of highly expressed samples and the absolute expression values. Regardless of datasets and analytic methods, we found that men have higher *ACE2* and *TMPRSS2* expression, which likely contributes to the sex difference in COVID-19 susceptibility. While inspecting individual organs, the kidney is among the top tissues with high expression of *ACE2* and *TMPRSS2*, and is the only tissue showing expression difference of *ACE2* and *TMPRSS2* in both datasets. We noted that *ACE2* expression presented a clear bimodal distribution in dataset G, but not in dataset A. This is because we removed samples with undetected expression in RNA-Seq processing (TPM < 0.5, datasets G and T) while kept all samples profiled in microarray processing (dataset A). Further inspection of these samples revealed a cluster of normal kidney samples has a lower expression of *ACE2*. When we kept the samples with higher expression of *ACE2* (microarray expression > 5) in G, we still observed higher expression of *ACE2* in men. Subsequent computational cell type enrichment analysis revealed that kidney mesangial cells are detected only in male COVID-19 patients’ blood and the creatinine levels are higher in male COVID-19 patients than in female COVID-19 patients. Because kidney disease is an important indicator of in-hospital mortality, we reasoned that the kidney is likely an organ accounting for sex differences in COVID-19, and the expression of *ACE2* might be a factor.

An additional difference resulted from host responses after the infection of SARS-CoV-2. In addition to reported immune response differences, we found vast differences in non-immune cells such as mitochondria functions, phagocytosis, and cholesterol biosynthesis, suggesting men and women have unique response trajectories after infection and latency. Of note, independent of sex, younger people (<=60) have much more differentially expressed genes than older people (>60); independent of age, women tend to have more differentially expressed genes than men. In both upper airway datasets, the subtle difference of host response between older male COVID-19 patients and older male healthy individuals might suggest the weakening responses to viral infection with increasing age, although the causal relation between host responses and disease susceptibility needs further exploration.

Hormone levels are an additional pillar of sex differences, although the difference in sex hormone effect is subtle in older people. Neither agonists nor antagonists of two hormone receptors ESR and AR displayed any activity against SARS-CoV-2 infection. One progesterone receptor agonist OHPC showed some activity, but another agonist desogestrel did not, suggesting OHPC may act through an off-target effect. One study demonstrates the efficacy of a group of sigma receptor inhibitors including OHPC against SARS-CoV-2 *in vitro* ^20^. Interestingly, a group of SERMs, acting as agonists, antagonists, or a mix of agonists and antagonists, exerted considerable activity *in vitro*. Prior studies found one SERM, toremifene, destabilizes a glycoprotein preventing fusion between the viral and endosome membranes in EBOVs ^22^. Our *in vitro* data showed neither increasing nor decreasing steroid hormone concentrations of estrogen, androgen, or progesterone exerted antiviral activity; however, it does not exclude the possibility of their benefits through other mechanisms such as anti-inflammatory ^28^.

Our work has the following limitations. First, in the quantitative analysis of *ACE2* differences, the batch effect between studies, variations among the three datasets, and other unknown confounding effects do exist; however, the main conclusions are robust, as we compared the samples that were produced using the same platform and processed under the same pipeline, used three relatively large independent datasets, and analyzed both categorical and continuous data. Second, when studying cell composition of bulk COVID-19 patient samples, although the computational tool Xcell we employed has been widely used, the exact cell fractions could be quantified more precisely using single-cell technologies. Furthermore, because of heterogeneous cell compositions in the samples, the DE genes might primarily reflect the difference of various cell types rather than that within infected cells. Lastly, the effect of sex hormone drugs ideally should be investigated in animal models or preferably in the clinic. The launched clinical trials are expected to provide more evidence soon. Nevertheless, our analysis of an extensive genomic, clinical, and drug-screening data quantitatively depicts sex differences in COVID-19 from three aspects.

## Methods

### Data collection

Treehouse OCTAD (T): We downloaded the processed TPM data and phenotype data from the Treehouse project ^14^. We used the same pipeline TOIL to process additional samples and integrated them into a single dataset OCTAD ^29^. This dataset has been used for drug repositioning. In this work, we took the subset of OCTAD where samples have tissue, sex, and age information. The subset includes samples from healthy normal, primary cancer, and adjacent normal tissue samples. This dataset has complete information of sex and age, as well as a fairly complete annotation of tissues. In Figure 1, we only analyzed healthy normal (GTEx) and adjacent normal samples (TCGA). Although healthy normal samples include some diseased patients and some samples are mixed with cancer cells, it remains the best resource for normal tissue samples.

ARCHS4 GPL11154 (A): We downloaded transcript TPM data from the ARCHS4 project which harmonized RNA-Seq sequence data from HiSeq 2000, HiSeq 2500, and NextSeq 500 platforms for human experiments from GEO and SRA. Reads were aligned with Kallisto using a custom cloud computing platform. Human samples were aligned against the GRCh38 human reference genome. The integrated expressions allowed comparing gene expression across tissues in ARCHS4. The metainformation of these samples was downloaded from ARCHS4, and regular expression pattern matching was employed to assist in tissue and age labeling. Only high confidence predictions of tissue and age were considered as labeled data for building machine learning models. We observed a large variation of expression among the three platforms; to facilitate subsequent analysis, we decided to choose HiSeq 200, which is the earliest and most commonly used platform. We drew similar conclusions from the other two platforms, which were not included in the analysis.

GEO GPL570 (G): GPL570 is the most popular microarray platform. CEL files from the GPL570 platform were downloaded using GEOquery. A total of 165 corrupted CEL files were removed from the analysis. Due to size and computational resources, GPL570 was divided into 42 batches (Average: 3477 CELs per batch). Each batch was then normalized with the Affy package using RMA. Selected batches were normalized with justRMA to maintain large batch size. Median was used to merge expression of multiple probes. We included all the samples profiled under this platform. The dataset covers human tissue sample, cultured human cell samples, and those treated with various perturbagens. In order to perform unbiased analysis, we did not exclude cell line samples frequently used at the bench. Sample metadata such as title, source name, and characteristics were pulled out from GEOmetadb.

### Meta-data curation and imputation

For Treehouse OCTAD (T), since sex and age information were relatively complete, we used only labeled data to perform the analysis, which also served as a reference for other datasets. For ARCHS4 GPL11154 (A) and GEO GPL570 (G), only less than 1/3 samples were labeled (Table S1). For samples taken from sex-specific tissues such as prostate and testis, we manually imputed their sex as male. However, the missing rate was still high after this process. Therefore, we further utilized state-of-the-art machine learning models to impute missing labels. Given a dataset, for each target of interest, i.e., sex, age, or tissue, we extracted the gene expression of target-specific (enriched) genes ^30,31^ for all the samples and used labeled data to build a prediction model. For sex and tissue, we built a multi-task deep neural network ^32^ (Figure S7) to predict them together since sex and some tissues are dependent on each other (e.g., only males have prostate/testis samples while breast samples are more likely from females; building models independently for sex and tissue may result in futile predictions such as female-testis). For age prediction, when adding age prediction into a multi-task learning framework, sex and tissue tasks were dominant over age prediction which yielded unsatisfactory results. Therefore, we treated age prediction as a single task and used the extreme gradient boosting (XGBoost) ^33^ to build the model. All the hyper-parameters were tuned using 5-fold cross-validation and the detailed experiment settings can be found in Supplementary Text. After the classifiers were built, we applied it to unlabeled data and predicted their labels. Only predictions with high confidence were kept for later use. For sex, we considered prediction with probability of <0.1 (female) and >0.9 (male) as confident. For multi-class targets (age: 3 classes; tissue: 14 classes), we considered class probability > 0.6 as confident. After that, human inspections were also involved to correct the potentially mislabeled samples based on the characteristics of each sample from the raw source file. Age prediction using gene expression data remains challenging, especially with the context of disease states, so we only predicted three age groups (0–19, 20–59, and >60). We observed that ages 40s and 50s are more likely to be misclassified. In the analysis, we only chose highly confident predictions and focused on the comparison of the younger and older group. In summary, we explored multiple options to ensure that each step produces optimal results. The complete labeled percentage for sex, age, and tissue before and after imputation can be found in Table S1. The model accuracy for all the tasks across all datasets can be found in Table S3, and the visualizations for learned representations are shown in Figure S8.

### Clinical data analysis

Using the Mount Sinai Data Warehouse, we compiled de-identified electronic medical records (EMR) data including age, sex, race, and creatinine levels for inpatients and outpatients confirmed with COVID-19 at Mount Sinai Health System up to 05/10/2020. We normalized the creatinine levels by using x/1.2 for males and x/1.1 for females (x was the creatinine level of the corresponding patient) since the reference range for males is 0.6~1.2 mg/dL and for females is 0.5~1.1 mg/dL. For logistic regression, we set the normalized creatinine > 1 as 1 for both in- and outpatients. In logistic regression, we set sex-female and race-white as the reference. Patient summary statistics can be found in Extended Data and more characteristics of the patients were reported elsewhere ^34,35^.

### Statistics and data analysis

For the comparison of sex differences in the proportion of samples with high expression of virus entry proteins, we employed logistic regression with the adjustment of age, tissue, and data source whenever possible. Since smoking status is emerging as an inconclusive factor accounting for sex differences in COVID-19, we adjusted smoking status in a small set (non-smoker: 859, smoker: 1,542), and found men still have higher *ACE2* expression than women (data not shown). Because of the limited size, we did not include smoking status in the main analysis. In tissue analysis, we chose 14 main tissues based on their sample counts and their importance; unknown refers to samples either belonging to other tissues or with low-confidence tissue prediction.

The expression of virus entry proteins is not normally distributed across samples: a majority of them with undetectable expression (TPM < 0.5), and a considerable number of samples with high expression of these entry proteins. We chose the top 10% most highly expressed samples as the high group (label 1) and the remaining as the normal group (label 0). We also explored the thresholds 75%, 80%, 85%, and 95% (the TPM is less than 0.5 at the threshold of 75%). There are variations of ORs among these thresholds, but the conclusions did not change (Figure S9). Such grouping enabled the incorporation of three datasets, the inclusion of samples with undetectable expression, and the mitigation of batch effect between studies. In addition, we applied a similar analysis to individual studies (i.e., GEO GSEs) (Supplementary Text).

The sex difference in continuous gene expression of target genes was evaluated by two metrics: the ratio of average gene expression between female and male groups and the difference of median gene expression between female and male groups. The 95% CI of ratio estimate between female and male groups is obtained by bootstrapping: sampling with replacement and calculating the ratio of average gene expression between female and male for 1000 times, with 2.5% and 97.5% quantile recorded. The difference of median gene expression between women and men is computed using a two-sided Wilcoxon rank-sum test with 95% CI and p-value reported. Since low abundance genes in RNA-Seq samples (TPM < 0.5) are often noisy, these samples were removed accordingly in Figure 1 and Figure S2.

### COVID-19 patient sample processing

We searched GEO and SRA using keywords “COVID” and “SARS-CoV-2” and chose those datasets with >3 samples in each sex group (e.g., male healthy, male COVID). We ended with four datasets in the following analysis (Table S4). Two datasets were from upper airways, one dataset was from PMBC and one was from leucocytes. The dataset from leucocytes includes patient severity (ICU vs. non-ICU). Reprocessed raw sequence data available at SRA were downloaded and mapped to the human Hg38 transcriptome using the ENSEMBL GRCh38.p3 annotation using STAR aligner (https://github.com/alexdobin/STAR). The read count mapped on transcriptome was used for DE analysis. All possible comparisons between male and female samples (female-CT vs. male-CT, female-SARS-Cov2 vs. male-SARS-cov2, female-CT vs. female-SARS-Cov2, and male-CT vs. male-SARS-Cov20) were performed. The absolute log2foldchange >=1 with a false discovery rate < 0.01 computed from the EdgeR ^36^ package was chosen to identify DE genes. The DE genes of these comparisons were further compared via vennDiagram R package to find out common genes and specific genes for each comparison. Furthermore, DE genes specific to Control vs. SARS-cov2 in male and Control vs. SARS-cov2 in female were applied to identify enriched pathways through the enrichR API ^37^. The sequence alignment, DE computation, and pathway enrichment were implemented in the OCTAD package ^29^. XCell was employed to infer cell composition ^19^.

### In vitro drug testing

#### Virus and Cells

Vero cells were obtained from the American Type Culture Collection (ATCC CCL-81; Manassas, VA, USA) and maintained at 37°C with 5% CO_2_ in Dulbecco’s Modified Eagle’s Medium (DMEM; Welgene, Gyeongsan, Republic of Korea), supplemented with 10% heat-inactivated fetal bovine serum (FBS) and 1X Antibiotic-Antimycotic solution (Gibco/Thermo Fisher Scientific, Waltham, MA, USA). Calu-3 used in this study is a clonal isolate, which shows higher growth rate compared to the parental Calu-3 obtained from the American Type Culture Collection (ATCC CCL-81). Calu-3 was maintained at 37°C with 5% CO_2_ in Eagle’s Minimum Essential Medium (EMEM, ATCC), supplemented with 20% heat-inactivated fetal bovine serum (FBS), 1X MEM-NEAA (Gibco) and 1X Antibiotic-Antimycotic solution (Gibco). SARS-CoV-2 (βCoV/KOR/KCDC03/2020) was provided by Korea Centers for Disease Control and Prevention (KCDC), and was propagated in Vero cells. Viral titers were determined by plaque assays in Vero cells. All experiments using SARS-CoV-2 were performed at Institut Pasteur Korea in compliance with the guidelines of the KNIH, using enhanced Biosafety Level 3 (BSL-3) containment procedures in laboratories approved for use by the KCDC.

#### Reagents

All compounds were purchased from MedChemExpress (Monmouth Junction, NJ), and dissolved in DMSO. Anti-SARS-CoV-2 N protein antibody was purchased from Sino Biological Inc. (Beijing, China). Alexa Fluor 488 goat anti-rabbit IgG (H + L) secondary antibody and Hoechst 33342 were purchased from Molecular Probes. Paraformaldehyde (PFA) (32% aqueous solution) and normal goat serum were purchased from Electron Microscopy Sciences (Hatfield, PA) and Vector Laboratories, Inc. (Burlingame, CA), respectively.

#### Dose-response curve (DRC) analysis by immunofluorescence

Ten-point DRCs were generated for each drug. Vero cells were seeded at 1.2 × 10^4^ cells per well in DMEM, supplemented with 2% FBS and 1X Antibiotic-Antimycotic solution (Gibco/Thermo Fisher Scientific). Calu-3 cells were seeded at 2.0 × 10^4^ cells per well in EMEM, supplemented with 20% FBS, 1X MEM-NEAA (Gibco) and 1X Antibiotic-Antimycotic solution (Gibco) in black, 384-well, μClear plates (Greiner Bio-One), 24 h prior to the experiment. Ten-point DRCs were generated, with compound concentrations ranging from 0.1–50 μM. For viral infection, plates were transferred into the BSL-3 containment facility and SARS-CoV-2 was added at a multiplicity of infection (MOI) of 0.05 or 0.1 for Vero and Calu-3 cells, respectively. The cells were fixed at 24 hpi with 4% PFA and analyzed by immunofluorescence. The acquired images were analyzed using in-house software to quantify cell numbers and infection ratios, and antiviral activity was normalized to positive (mock) and negative (0.5% DMSO) controls in each assay plate. DRCs were generated in Prism7 (GraphPad) software, with Dose-response-inhibition nonlinear regression analysis. IC_50_ and CC_50_ values were obtained with the identical analysis method. Mean values of independent duplicate experiments were used for analysis. Each assay was controlled by Z’-factor and the coefficient of variation in percent (%CV).

## Supplementary Material

Supplement

## Figures and Tables

**Table 1.** Odds ratios of sex in the prediction of the high expression group in the Healthy dataset and in the Merged dataset. In the regression model, Y is the binary expression level of an entry protein, and X is sex with female being the reference. The all age group is adjusted for age, tissue, and data source, and the older group (>60 years) is adjusted for tissue and data source.

	Samples in the Healthy dataset		Samples in the Merged dataset	
	all ages (n=8,066)	>60 (n=2,849)	all ages (n=220,835)	>60 (n=37,911)
ACE2	1.06 [0.86–1.32]	1.02 [0.72–1.44]	1.25 [1.19–1.30]*	1.15 [1.07–1.23]*
TMPRSS2	1.03 [0.85–1.27]	0.91 [0.65–1.29]	1.28 [1.23–1.34]*	1.32 [1.24–1.42]*
ACE2 & TMPRSS2	0.80 [0.52–1.24]	0.55 [0.27–1.10]	1.16 [1.09–1.24]*	1.12 [1.03–1.22]#

*: p < 0.001 and

#: 0.009.

**Table 2:** *In vitro* anti-SARS-CoV-2 efficacy of steroid sex hormone drugs. The IC_50_s in Vero and Calu-3 cells were summarized from dose-response curves (Figure S6). The IC_50_s of SERMs (Selective Estrogen Receptor Modulators) were collected from published studies (Extended Data).

Drug	Drug class	Vero (uM)	Calu-3 (uM)	Other studies (uM)
Estradiol	Estrogen receptor agonist	>50	27.3	
Fulvestrant	Estrogen receptor antagonist	>50	>50	
Danazol	Androgen receptor agonist	>50	25.6	
Bicalutamide	Androgen receptor antagonist	>50	>50	
Hydroxyprogesterone caproate	Progesterone receptor agonist	13.0	6.4	
Desogestrel	Progesterone receptor agonist	>50	>50	
Bazedoxifene	SERM	NA	NA	3.4 ^24^
Droloxifene	SERM	NA	NA	6.6 ^24^
Ospemifene	SERM	NA	NA	12.6 ^21^
Raloxifene hydrochloride	SERM	NA	NA	2.2 ^21^
Tamoxifen	SERM	NA	NA	9.0 ^25^
Toremifene	SERM	NA	NA	11.3 ^25^

## Data Availability

Source data and code is available at GitHub (https://github.com/Bin-Chen-Lab/covid19_sex).
