# Sex differences in viral entry protein expression and host transcript responses to SARS-CoV-2

**DOI:** 10.21203/rs.3.rs-100914/v2

**Published:** 2020-12-31

**Authors:** Mengying Sun, Rama Shankar, Meehyun Ko, Christopher Daniel Chang, Shan-Ju Yeh, Shilong Li, Ke Liu, Guoli Zhou, Jing Xing, Austin VanVelsen, Tyler VanVelsen, Shreya Paithankar, Benjamin Y. Feng, Krista Young, Michael Strug, Lauren Turco, Zichen Wang, Eric Schadt, Rong Chen, Xiaohong Li, Tomiko Oskotsky, Marina Sirota, Benjamin S. Glicksberg, Girish N. Nadkarni, Adam J. Moeser, Li Li, Seungtaek Kim, Jiayu Zhou, Bin Chen

**Affiliations:** 1. Department of Pediatrics and Human Development, Michigan State University, Grand Rapids, Michigan, USA.; 2. Department of Computer Science and Engineering, Michigan State University, East Lansing, Michigan, USA.; 3. Department of Pharmacology and Toxicology, Michigan State University, Grand Rapids, Michigan, USA.; 4. College of Osteopathic Medicine, Michigan State University, East Lansing, Michigan, USA.; 5. Biomedical Research Informatics Core, Clinical & Translational Sciences Institute, Michigan State University, East Lansing, Michigan, USA.; 6. Van Andel Research Institute, Grand Rapids, Michigan, USA.; 7. Sema4, Stamford, CT, Connecticut, USA.; 8. Department of Genetics and Genomic Sciences, The Icahn Institute for Genomics and Multiscale Biology, Icahn School of Medicine at Mount Sinai, New York, USA.; 9. Department of Obstetrics and Gynecology, Spectrum Health, Grand Rapids, Michigan, USA; 10. Department of Obstetrics, Gynecology and Reproductive Biology, Michigan State University, Grand Rapids, Michigan, USA; 11. Emergency Medicine Residency, Spectrum Health, Grand Rapids, Michigan, USA; 12. Department of Electrical Engineering, National Tsing Hua University, Hsinchu, Taiwan; 13. Zoonotic Virus Laboratory, Institut Pasteur Korea, Seongnam, Korea; 14. Large Animal Clinical Sciences and Department of Physiology, Michigan State University, East Lansing, Michigan, USA.; 15. Department of Pediatrics and Bakar Computational Health Sciences Institute, University of California, San Francisco, CA, USA; 16. Department of Medicine, Icahn School of Medicine at Mount Sinai, New York, NY, USA; The Hasso Plattner Institute of Digital Health at Mount Sinai, Icahn School of Medicine at Mount Sinai, New York, NY, USA; The Charles Bronfman Institute of Personalized Medicine, Icahn School of Medicine at Mount Sinai, New York, NY, USA.; 17. The Hasso Plattner Institute of Digital Health at Mount Sinai, Icahn School of Medicine at Mount Sinai, New York, NY, USA; Department of Genetics and Genomic Sciences, Icahn School of Medicine at Mount Sinai, New York, NY, USA.

**Keywords:** sex difference, COVID-19

## Abstract

Epidemiological studies suggest that men exhibit a higher mortality rate to COVID-19 than women, yet the underlying biology is largely unknown. Here, we seek to delineate sex differences in the gene expression of viral entry proteins ACE2 and TMPRSS2, and host transcriptional responses to SARS-CoV-2 through large-scale analysis of genomic and clinical data. We first compiled 220,000 human gene expression profiles from three databases and completed the meta-information through machine learning and manual annotation. Large scale analysis of these profiles indicated that male samples show higher expression levels of *ACE2* and *TMPRSS2* than female samples, especially in the older group (>60 years) and in the kidney. Subsequent analysis of 6,031 COVID-19 patients at Mount Sinai Health System revealed that men have significantly higher creatinine levels, an indicator of impaired kidney function. Further analysis of 782 COVID-19 patient gene expression profiles taken from upper airway and blood suggested men and women present distinct expression changes. Computational deconvolution analysis of these profiles revealed male COVID-19 patients have enriched kidney-specific mesangial cells in blood compared to healthy patients. Together, this study suggests biological differences in the kidney between sexes may contribute to sex disparity in COVID-19.

A growing body of epidemiological evidence suggests that men and women are equally likely to contract the new coronavirus while men exhibit a higher mortality rate to COVID-19 than women ^1–3^. The analysis of 34 million cases and 1 million deaths across 99 countries provided by Globe Heath 50/50 2020 found that, for every 10 female COVID-19 cases, there are 10 cases and 14 deaths in men (as of 30 November) ^4^. According to the report from the Centers for Disease Control and Prevention (CDC), men account for 54.2% of the 261,530 total deaths (as of December 2020). Such sex disparity exists in all age groups ^5^. Sex-specific clinical characteristics analysis of patients in Wuhan, China showed that men with comorbidities presented a higher risk of being critically ill than men without comorbidities ^6^, and a similar analysis of patients in Urban New York found that chronic kidney disease is higher in deceased men and obesity is higher in deceased women ^3^. The compelling epidemiological evidence of sex disparity in COVID-19 and the urgency of this pandemic propelled the investigation of sex steroid hormone drugs in clinical trials (Estradiol: NCT04359329, Progesterone: NCT04365127, Degarelix: NCT04397718), although more preclinical evidence is needed. To underpin the biology of sex differences, hypotheses pertaining to the expression of viral entry proteins and immune systems have been actively explored ^7,8^.

SARS-CoV-2 engages the receptor ACE2 (angiotensin-converting enzyme 2) for entry into the target cell through its spike protein ^9^. Its internalization requires priming of the spike protein by the cellular protease TMPRSS2 (transmembrane protease, serine 2) in the host cell ^10^, thus co-expression of ACE2 and TMPRSS2 on the target cell surface is required for virus entry. The high mortality in patients with COVID-19 may be partially driven by the strong affinity of the virus to ACE2 and the facilitation from TMPRSS2. For example, the higher expression of *ACE2* in the nasal epithelium of children compared to adults may be linked to a lower case rate in children ^11^. Plasma concentrations of ACE2 were observed to be higher in men than in women in cohorts of patients with heart failure ^12^ and higher expression of *ACE2* and *TMPRSS2* was also observed in men with asthma ^13^. To perform tissue-wide expression analyses of *ACE2* and/or *TMPRSS2* between sexes, a few studies ^14–16^ exploited bulk or single-cell RNA-Seq samples primarily profiled from healthy individuals. However, many of the hospitalized COVID-19 patients have an underlying illness which increases mortality risks, and are in older age range (>60 years) ^17^. Furthermore, the number of patients in these studies ^14–16^ was relatively small.

Sex differences in immune responses in COVID-19 were examined in a recent study, where blood samples from female patients were found to present more robust T-cell activation than male patients during SARS-CoV-2 infection ^18^. Transcriptomic comparison of samples from nasopharyngeal swabs revealed that male patients had reduced B cell-specific and NK cell-specific transcripts and an increase in inhibition of nuclear factor kappa-B signaling ^19^. Neutralizing autoantibodies that neutralize the ability of the corresponding type I IFNs to block SARS-CoV-2 infection were also found to express more in old men ^20^. In addition to the postulation of immune responses, the genomic profiles produced in these studies and others offer a great opportunity to perform a holistic examination of the difference of cellular responses to the virus.

In this study, we repurpose existing large gene expression profiles to examine sex differences before and after SARS-CoV-2 infection. First, we leverage public gene expression profiles covering a wide range of ages, tissues, and disease conditions, to quantify expression differences of virus entry proteins, and later utilize Electronic Medical Records (EMR) to validate findings. Next, we harness the emerging COVID-19 patient gene expression profiles to characterize transcriptional response differences between sexes in the upper airway and blood.

## Expression of ACE2 and TMPRSS2 in a diverse and comprehensive set of human samples

**Figure 1.**
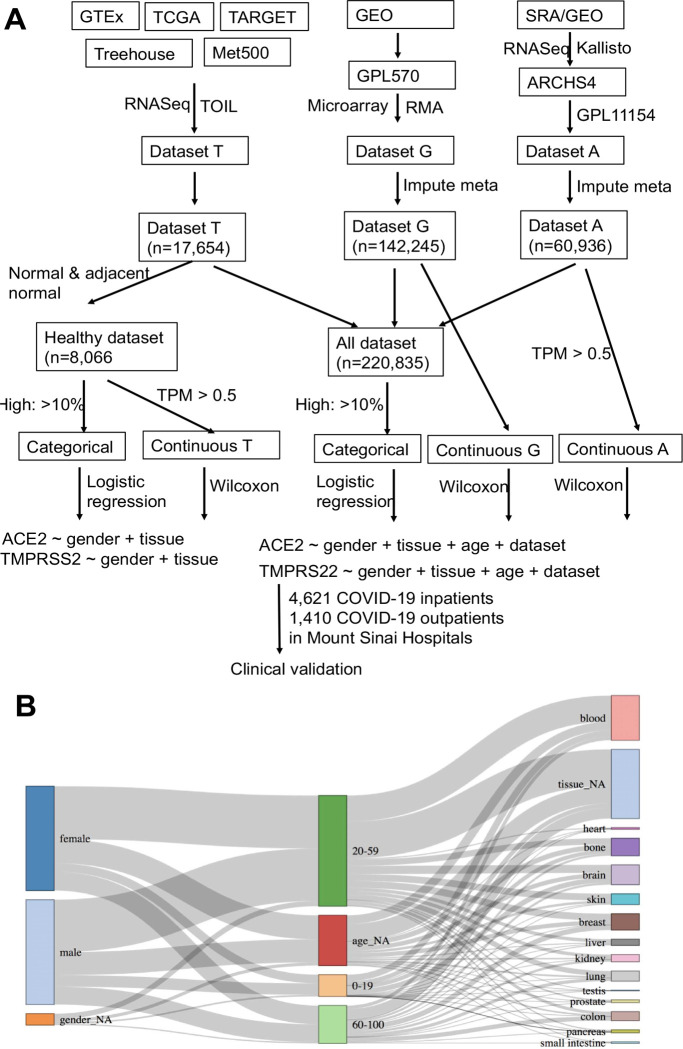
Large-scale analysis of expression differences in virus entry proteins. (**A**) Data collection and analysis workflow. We defined high-expression as the top 10% within individual datasets (T, G, A) and explored other thresholds as well. For T and A, samples with Transcripts Per Million (TPM) < 0.5 were removed in order to reduce technical noises. ARCHS4: All RNA-seq and CHIP-Seq Signature Search Space developed by Ma’ayan Laboratory; GEO: Gene Expression Omnibus; GTEx: The Genotype-Tissue Expression; Kallisto: program for quantifying abundances of transcripts from bulk and single-cell RNA-Seq data; Meta500: 500 metastatic cancers; RMA: Robust Multi-array Average; SRA: Sequence Read Archive; TARGET: The Therapeutically Applicable Research to Generate Effective Treatments; TCGA: The Cancer Genome Atlas; TOIL: A RNASeq processing pipeline developed by UC Santa Cruz; Treehouse: Treehouse Childhood Cancer Initiative; TPM: Transcripts Per Million. (**B**) Sankey diagram of gender, age and tissue for the merged dataset. Predictions with low confidence remain as ‘NA’ after imputation (denoted as gender_NA, age_NA and tissue_NA in the diagram). The height of each box in a column represents the proportion of corresponding group in the dataset.

We first compiled three large independent expression datasets consisting of 220,835 samples from diverse tissue types and patient populations (healthy and disease conditions) and completed their meta-information, including sex, age group (younger: 0–19, middle: 20–59, and older: >60), and tissue of origin (14 main tissues), through machine learning and manual annotation (Figure 1). To minimize batch effects, all the samples in each dataset were profiled under the same platform and processed using the same pipeline. The first dataset was compiled from the Treehouse project (T), where 17,654 RNA-Seq samples primarily from consortium projects including TCGA, GTEx, and TARGET were processed through the Toil pipeline ^21^. The second dataset was downloaded from the ARCHS4 (A) project, where 60,936 human RNA-Seq samples profiled under the Illumina HiSeq 2000 platform were aligned using Kallisto ^22^. The last dataset was collected from the GEO (G), where 145,947 samples profiled under the Affymetrix GPL570 platform were processed using Robust Multi-array Average (RMA). The sex, age group, and tissue of origin were obtained from the original resources (Methods); however, a substantial number of samples had missing metadata, especially in datasets A and G, where only 1,407 and 4,392 samples have all sex, age, and tissue information, respectively. Leveraging their expression features, we built machine learning models (deep multi-task neural network and XGBoost) that completed metadata for the majority of samples with high confidence (Table S1). The average accuracy of the predictions in A and G are 93.6% (Sex), 80.8% (Age group), and 87.3% (Tissue) (Table S2). All of these predictions were further manually inspected based on unstructured sample meta-information available in the source files when possible. In total, we ended with 220,835 samples for the following analysis (Figure 1B, detailed distribution for individual datasets in Figure S1).

Since both the proportion of samples with high expression of entry proteins and the absolute expression value of these proteins within individual samples are important to understand sex differences, we analyzed both categorical and continuous expression data (Figure 1). We first merged all three datasets into one single matrix (referred to as the Merged dataset) consisting of 220,835 samples, after categorizing them into high (e.g., top 10% within each dataset) and normal expression groups (i.e., *ACE2* high *vs. ACE2* normal, *TMPRSS2* high vs. *TMPRSS2* normal, and *ACE2&TMPRSS2* high vs. *ACE2&TMPRSS2* normal) in individual datasets. The Merged dataset, including diseased samples, healthy samples, and those samples with the treatment of perturbagen samples, might be one of the best resources to investigate expression of entry proteins thus far. Similarly, we compiled a single matrix consisting of 8,066 healthy samples from T (referred to as the Healthy dataset). Logistic regressions were applied to predict the high expression group using age, tissue, and sex as features (by default, 95% confidence interval (CI), female as a reference). In addition to analyzing categorical expression data using the Merged dataset, we compared absolute expression between sex groups for each dataset separately.

**Figure 2.**
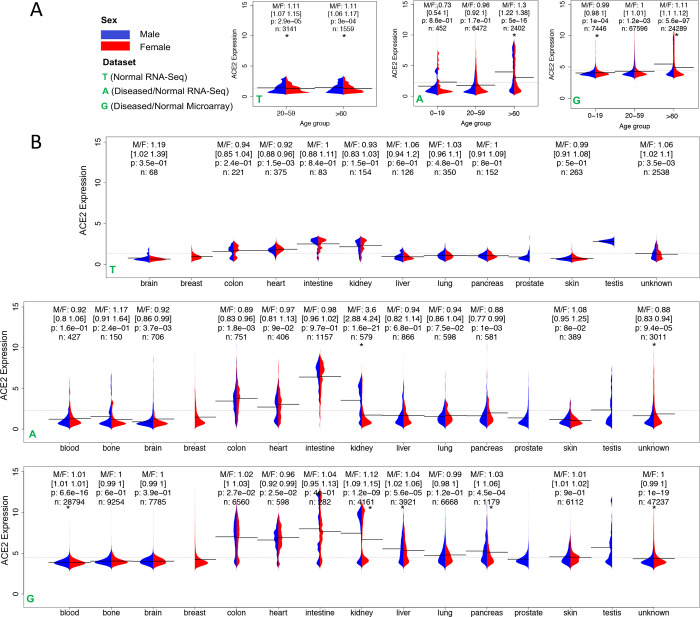
ACE2 expression across (**A**) age groups and (**B**) tissues in three datasets Treehouse (T), ARCHS4 (A), and GPL570 (G). In T, only normal and adjacent normal tissue samples were selected. Due to the limited sample size (n), the 0–19 age group, and the blood and bone tissues were not included in (A) and (B), respectively. M/F represents the ratio of mean expression between males and females. The 95% confidence interval of ratio was obtained using bootstrapping, i.e., sampling with replacement and calculating the ratio for 1000 times with 2.5% and 97.5% quantile reported. P-value was computed from a two-sided Wilcoxon rank-sum test on the difference of median expression between sexes. * indicates P < 0.001. The bar and the dashed line show the mean of expressions in each group and the mean of all expressions, respectively.

We did not observe any significant difference in the proportion of highly expressed *ACE2*, *TMPRSS2,* or *ACE2*&*TMPRSS2* samples between women and men in the Heathy dataset after adjusting for age and tissue (Table 1). However, the proportion of highly expressed samples in men is larger than in women in the Merged dataset (*ACE2*: OR 1.25 [1.19–1.30], P < 0.001; *TMPRSS2*: OR 1.28 [1.23–1.34], P < 0.001; *ACE2*&*TMPRSS2*: OR 1.16 [1.09–1.24], P < 0.001) (Table 1). In the older group, proportion difference was also observed in the Merged dataset (*ACE2*: OR 1.15 [1.07–1.23], P < 0.001; *TMPRSS2*: OR 1.32 [1.24–1.42], P < 0.001; *ACE2*&*TMPRSS2*: OR 1.12 [1.03–1.22], P=0.009), but not in the Healthy dataset (Table 1). Neither *ACE2* nor *TMPRSS2* is highly expressed in the majority of samples, while both are indeed highly expressed in a considerable number of samples in both men and women suggested by the long tails in both G and A, but not in T (normal) (Figure 2A). Compared to the younger group (0–19), the older group (>60) has a larger difference of *ACE2* expression between males and females (G: M/F 1.11 [1.1–1.12], P < 0.001 in older vs. M/F 0.99 [0.98–0.99], P < 0.001 in younger) (Figure 2A) as well as *TMPRSS2* expression (G: M/F 1.04 [1.03–1.04], P < 0.001 in older vs. M/F 1.0 [0.99–1.0], P=0.58 in younger) (Figure S2A). Further analysis of additional disease samples with the highest expression of *ACE2* revealed that Crohn’s disease, ulcerative colitis, Barrett’s esophagus, trachoma, and ichthyosis have overall higher *ACE2* expression in disease samples compared to control (Student’s t-test, P < 0.05, Figure S3). The small sample size for each disease obstructed the subsequent sex difference analysis. In summary, although expression difference of entry proteins between sexes was not observed in the Healthy dataset, higher *ACE2* expression was found in men, especially in older men, in the Merged dataset.

Due to expression variations in tissues, we next investigated whether expression differences are retained in individual tissues. Because of the wide coverage of samples in A and G, expression of *ACE2* has a larger variation in both datasets than in the Healthy dataset T, especially in the kidney, small intestine, heart, liver, and colon (Figure 2B), while a large variation exists in the expression of *TMPRSS2* in the kidney, small intestine, liver, colon, lung, pancreas, and prostate (Figure S2B). *ACE2* is not differentially expressed between sexes in the lung, an organ the viruses primarily attack (OR: 0.9 [0.78–1.04], P > 0.001), and women have even lower *TMPRSS2* expression in the lung in the Merged dataset [OR: 0.71(0.64–0.78), P < 0.001] (Table S3). Notably, the kidney is the only tissue showing a remarkable difference in *ACE2* expression between sexes in both A and G (Figure 2B). After adjusting for age and data source, the OR is 1.45 [1.26–1.67] (P < 0.001) (Table S3). The kidney is also the only tissue showing a significant difference in *TMPRSS2* expression between sexes in both A and G (Figure S2B).

**Figure 3:**
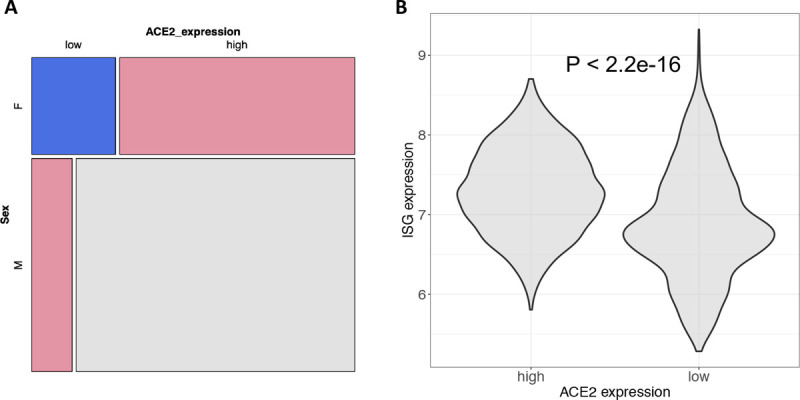
ACE2 expression in the kidney. (**A**) mosaic plot showing ACE2 expression differences between sexes in 2,033 kidney tissue samples with explicit information of disease status (1,654 kidney diseases and 379 normal tissues). Disease status was manually annotated based on sample characteristics provided in GEO. (**B**) correlation between ACE2 expression and ISG average expression. ISG genes were compiled based on the GO terms listed in Ziegler et al. 23. Wilcoxon rank-sum test was used to test the difference and GPL570 data were employed for both figures.

We were able to further map 28% of those samples with high *ACE2* expression to their diseases based on sample meta-information. The top mapped diseases are clear cell/renal cell carcinoma (60.8%), renal interstitial fibrosis (9.1%), acute kidney injury (7.5%), glomerulosclerosis (6.7%), nephritis (4.3%), and nephropathy (2.6%). Among 4,161 total kidney samples in G, 1,654 and 379 samples were explicitly indicated as kidney disease and normal kidney tissues in GEO, respectively. Using these new labeled samples, we repeated the analysis and confirmed that men have more number of samples with high *ACE2* expression than women (OR: 2.45 [1.89–3.18], P < 0.001) (Figure 3A). *ACE2* expression could be upregulated by type I INFs and *ACE2* is likely an interferon (IFN)-stimulated genes (ISG)^23^. We then computed average gene expression of all ISGs ^23^ in the kidney and observed that samples with high *ACE2* expression have higher ISG expression than those with low *ACE2* expression (p < 2.2e-16, Wilcoxon rank-sum test) (Figure 3B), suggesting the upregulation of *ACE2* in kidney might be associated with interferon activity.

Besides, steroid hormone receptors regulate the renin-angiotensin-aldosterone-system, where ACE2 is an essential component ^24^, we thus examined the expression relationship between ACE2 and steroid hormone receptors. *ACE2* expression has a higher correlation with *AR* expression (Androgen Receptor, Spearman Rho: 0.72, P < 0.001) than with ESR1 expression (Estrogen Receptor 1, Rho: 0.19, P < 0.001), *ESR2* expression (Estrogen Receptor 2, Rho: −0.12, P < 0.001), and *PGR* expression (Progesterone Receptor, Rho: 0.26, P < 0.001) in the kidney (Figure S4A). The genes regulated by AR also highly overlap with the genes positively co-expressed with *ACE2* in the healthy adrenal gland (P = 3.08E-5, Figure S4B), suggesting that *ACE2* expression might be associated with androgen receptor activity. Androgen signaling was identified to be a key modulator of ACE2 levels and treatment with antiandrogenic drugs reduced ACE2 expression and protected hESC-derived lung organoids against SARS-CoV-2 infection ^25^. We further evaluated the efficacy of estrogens, progesterones, and androgens against SARS-CoV-2 in Vero-6 and Calu 3 cells as well as surveyed the anti-SARS-CoV-2 activity of 62 steroid and non-steroid hormone drugs through literature search and querying of large-scale screening databases (Table S4, Figure S5). The protective role of any type of sex steroids including androgen inhibitors was not conclusive (Supplementary Materials). Due to the dramatic difference in hormone activities in vitro and in vivo, we decided to seek clinical evidence.

To find clinical evidence of these findings, we analyzed 6,031 COVID-19 patients (4,621 inpatients and 1,410 outpatients with available labs) for serum creatinine levels measured in five member hospitals at Mount Sinai Health System up to May 10, 2020. We observed that men have significantly higher serum creatinine levels than women after normalizing to sex-specific reference ranges and adjusting for age and race (Inpatients OR: 1.89 [1.66–2.15], P < 0.001; Outpatients OR 2.12 [1.68–2.66], P < 0.001) (Extended Data), indicating COVID-19 male patients are most likely to have kidney dysfunction than female patients. Whereas, both expression and clinical data analysis suggest that sex difference in the kidney is not specific to the older group (Table S3 and Extended Data). Recent studies reported that acute kidney injury is common in patients hospitalized with COVID-19 and is associated with increased mortality ^26,27^. Our accompanying study indicates chronic kidney disease is more common in men than in women who died from COVID-19 ^3^. Together, the expression difference of entry proteins in the kidney between sexes might be a factor contributing to sex differences in COVID-19 susceptibility.

## Sex stratified analysis of host responses to SARS-CoV-2

After contracting the virus, individuals may have varying responses to the infection. We then sought to dissect the differences through leveraging the emerging COVID-19 patient profiles. We searched GEO and SRA to obtain COVID-19 patient RNA-Seq samples and reprocessed raw sequence data when possible. We compiled four datasets with gender information (one from upper airway nasopharyngeal, one from upper airway naso/oro-pharyngeal, one from blood PBMC, and one from blood leukocytes), totaling 782 samples (Table S5). In each dataset, the ratio of the number of samples between sexes is close to 1. For each large upper airway dataset, we stratified samples into an older age group (>60 years) and a middle age group (20–60 years). We enumerated all the possible comparisons for each dataset (i.e., female control vs. female patient, male control vs. male patient, female control vs. male control, and female patient vs. male patient), with each comparison using the same thresholds to select differentially expressed genes.

In comparing female and male samples either in the control group or in the COVID-19 group, only a few sex-specific genes were dysregulated between women and men. Neither *ACE2* nor *TMPRSS2* was differentially expressed between sexes in either group. As SARS-CoV-2 likely infects nasal epithelial cells to enter into the body ^28^, the comparable expression of entry proteins in the samples taken from nasopharyngeal swab between sexes may explain that men and women are equally likely to contract the new coronavirus.

**Figure 4.**
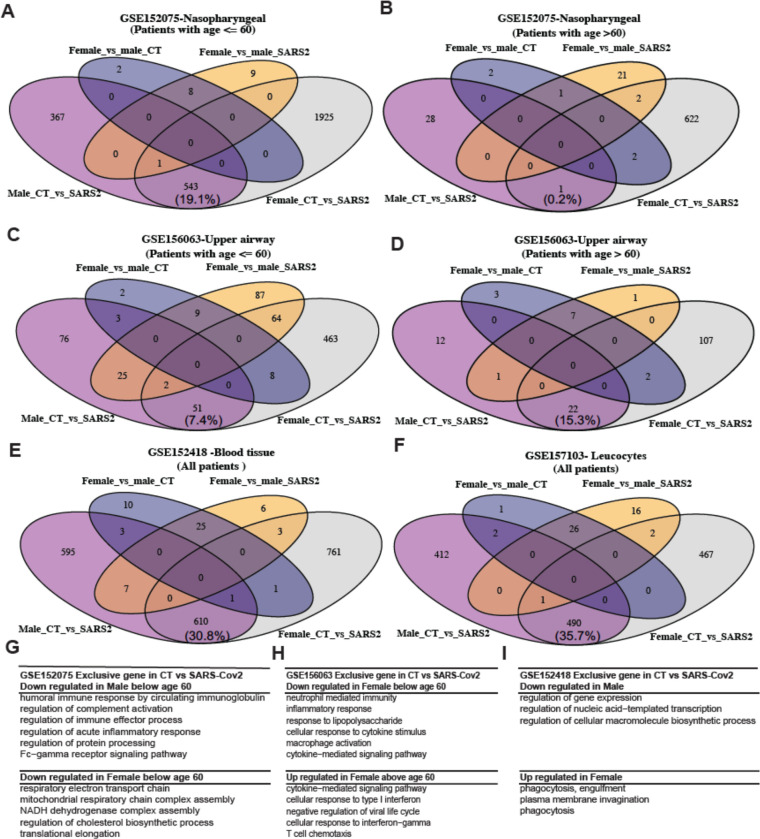
Host responses to SARS-CoV-2 in upper airway and blood. Differentially expressed (DE) genes in different comparisons (Male CT vs. SARS2, Female CT vs. SARS2, Female CT vs. Male CT, and Female SARS2 vs. Male SARS2) across multiple datasets: (**A**) from upper airway at age <= 60 years, (**B**) from upper airway at age >60 years, (**C**) from upper airway at age <= 60 years, (**D**) from upper airway at age >60 years, (**E**) from blood PBMC and (F) from blood leucocytes. Enriched biological processes of sex-specific genes (i.e., Male_CT_vs_SARS2, Female_CT_vs_SARS2): (**G**) from nasopharyngeal, (**H**) from upper airway, and (**I**) from blood leukocytes. Fold change > 2 and adjusted p-value < 0.001 were used to select DE genes and adjusted p-value < 0.1 was used to identify significantly enriched GO biological processes. Those DE gene sets without any significant GO terms were not shown. Patients were stratified into two groups (<= 60 and > 60) whenever age information was available and sample size in each group is greater than 10. The percentage of common DE genes was computed as the ratio between the number of common DE genes and the total number of distinct genes (Male_CT_vs_SARS2 plus Female_CT_vs_SARS2).

However, expression of a vast number of genes was significantly changed (p < 0.001, absolute fold change > 2) between healthy patients and COVID-19 patients in either men or women (e.g., 4269 DE in female CT vs. SARS2 and 911 DE in male CT vs. SARS2 in middle age group; 627 DE in female CT vs. SARS2 and 29 DE in male CT vs. SARS2 in older age group in GSE152075) (Figure 4 A–F). Interestingly, such changes seem unique to each sex with only a small portion of DE genes shared by both sexes. The two datasets from blood show the largest number of shared DE genes (35.7% and 30.8%, Figure 4E, 4F), while the dataset from older male upper airway has the lowest number of shared DE genes (0.1%, Figure 4B). The lower number is likely because fewer genes were differentially expressed in older male upper airways after SARS-CoV-2 infection. Female patients presented very distinct gene expression changes in all datasets, especially in the younger group (Figure 4A, 4C). Pathway enrichment analysis of these distinct DE genes confirmed the immune response differences (cytokinin mediated signaling, cellular response to interferon-gamma and interferon 1) as previously reported ^18,19^; however, a few other non-immune related pathways were enriched in female patients, including down-regulation of mitochondrial respiratory responses and regulation of cholesterol biosynthesis (Figure 4 G–I). The younger male group presented the downregulation of various immune responses such as humoral immune response, acute inflammatory response, and Fc- gamma signaling pathways (Figure 4G), while the older male group had no enriched pathways. Together with the higher susceptibility in older men, the analysis suggests that men and women have distinct host transcriptional responses in addition to immune responses. Importantly, weak host responses in the upper airway could be one indicator of susceptibility.

**Figure 5:**
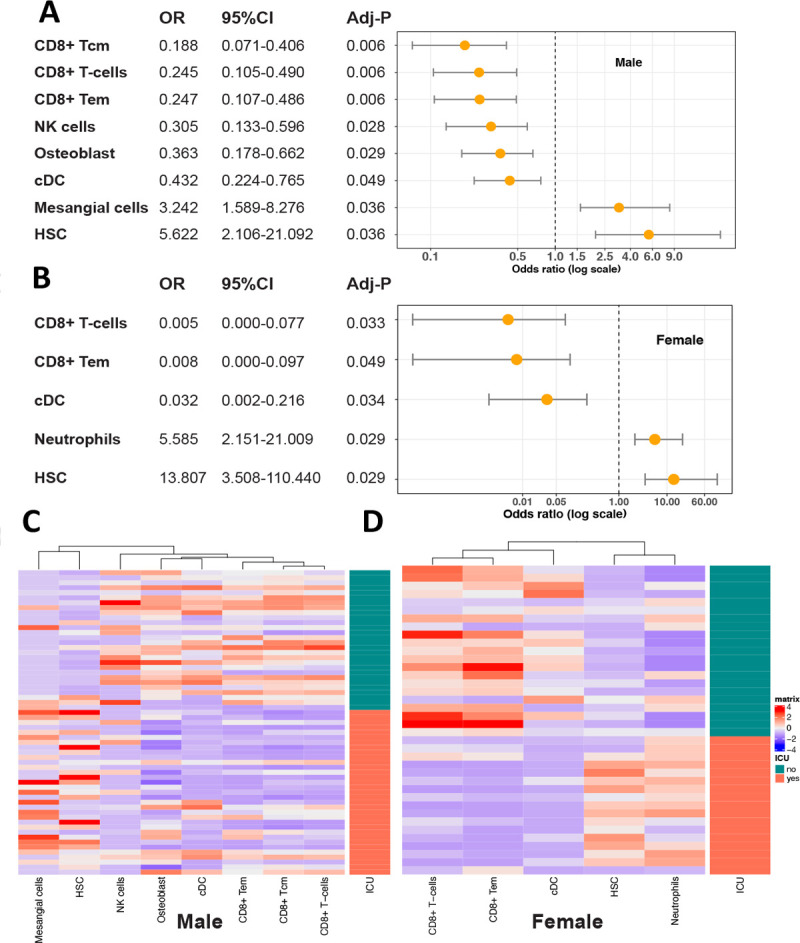
Odds ratio (OR) and enrichment of cells in COVID-19 patients. Odds ratios of different cells between ICU and non-ICU patients (**A**) in male and (**B**) in female. Logistic regression was performed with non-ICU patients as the reference and age adjusted. P values were corrected by a multiple hypothesis test and cell types with an adjusted p value <= 0.05 were selected. Heatmaps show the enrichment value of cells in male (**C**) and (**D**) in female.

We further inferred the enrichment of 64 cell types in COVID leucocytes using Xcell ^29^ and compared cell type enrichment. In both men and women, CD8+ T-cells and memory CD8 T Cell were suppressed in COVID ICU patients (Figure 5A, 5B). NK cells were suppressed in male ICU patients while neutrophils were elevated in female ICU patients. One striking difference between men and women came from the enrichment of mesangial cells, a kidney-specific cell type (control vs. COVID: male p-value of 1E-7 and female p-value of 1E-1, Student’s t-test). Logistic regression analysis of enrichment of mesangial cells with disease severity (non-ICU vs. ICU) indicated patients with higher enrichment of mesangial cells are more likely admitted into ICU [OR: 3.2(1.6–8.3), P < 0.001] (adjusted for age, Figure 5B). The enrichment of mesangial cells was detected in a substantial number of ICU COVID-19 blood, although the absolute value is low (Figure 5C, 5D). Together with the higher expression of *ACE2* and higher creatine levels in men, this analysis implies that impaired kidney function could be one source of sex differences.

## Discussion

COVID-19-related death is mainly associated with being male, older age, and comorbidities ^30^. The virus first enters the nose and throat, and then travels down to attack lung, which likely causes substantial respiratory pathology including acute respiratory distress syndrome. Its reach can extend to many other organs like blood vessels, liver, kidney, heart, and brain ^31^. ACE2 and its partner proteins are the key facilitators of virus entry into different organs. Therefore, their expression levels could be associated with disease severity and further mortality, and their expression differences between sexes could partially explain the higher mortality in men. Previous efforts did not detect the difference ^14,15^, likely because only healthy tissues were examined. In our Healthy dataset, we did not observe the difference either. Therefore, a comprehensive dataset covering a wide range of tissue samples including diseased samples is necessary to solve the puzzle.

In order to draw robust conclusions, we utilized two large independent datasets based on distinct technologies: microarray and RNA-Seq. In addition, we examined the differences according to the percentage of highly expressed samples and the absolute expression values. Regardless of datasets and analytic methods, we found that men have higher *ACE2* and *TMPRSS2* expression, which likely contributes to the sex difference in COVID-19 susceptibility. While inspecting individual organs, the kidney is among the top tissues with high expression of *ACE2* and *TMPRSS2*, and is the only tissue showing expression difference of *ACE2* and *TMPRSS2* in both datasets. We noted that *ACE2* expression presented a clear bimodal distribution in dataset G, but not in dataset A. This is because we removed samples with undetected expression in RNA-Seq processing (TPM < 0.5, datasets G and T) while kept all samples profiled in microarray processing (dataset A). The undetected transcripts in RNA-Seq could be either physiological or technical artifacts; therefore, we intended to remove them. The consistency of results from multiple platforms and multiple analytic methods confirms the removal is reasonable. Further inspection of these samples revealed a cluster of normal kidney samples has a lower expression of *ACE2*. When we kept the samples with higher expression of *ACE2* (microarray expression > 5) in G, we still observed higher expression of *ACE2* in men. Other than in the kidney, *ACE2* is not differentially expressed between sexes in other tissues including the nasopharynx and lung. It is not differentially expressed in the blood and upper airway in the COVID-19 patients, neither. We found old men do not exert profound responses to infection in airway. One recent study showed that old men do not present neutralizing autoantibodies that neutralize the ability of the corresponding type I IFNs to respond to the virus ^20^. Subsequent computational cell type enrichment analysis revealed that kidney mesangial cells are detected only in male COVID-19 patients’ blood and the creatinine levels are higher in male COVID-19 patients than in female COVID-19 patients. Together, we postulate that the virus may travel down to infect the cells with high expression of entry proteins in the kidney, resulting in the dysfunction of this organ. Because kidney disease is an important indicator of in-hospital mortality, we reasoned that the kidney is likely an organ accounting for sex differences in COVID-19, and the expression of *ACE2* might be a factor.

An additional difference resulted from host responses after the infection of SARS-CoV-2. In addition to reported immune response differences, we found vast differences in non-immune cells such as mitochondria functions, phagocytosis, and cholesterol biosynthesis, suggesting men and women have unique response trajectories after infection and latency. Both large-scale genome-wide CRISPR screenings confirmed that cholesterol synthesis is a key host response factor and targeting cholesterol synthesis using small molecules like 27-hydroxycholesterol could reduce SARS-CoV-2 infection in vitro ^32–34^. Of note, independent of sex, younger people (<=60) have much more differentially expressed genes than older people (>60); independent of age, women tend to have more differentially expressed genes than men. In two independent upper airway datasets, the subtle difference of host response between older male COVID-19 patients and older male healthy individuals might suggest the attenuated transcriptional responses to viral infection with increasing age, although the causal relation between host responses and disease susceptibility needs further exploration.

Our work has the following limitations. First, in the quantitative analysis of *ACE2* differences, the batch effect between studies, variations among the three datasets, and other unknown confounding effects do exist; however, the main conclusions are robust, as we compared the samples that were produced using the same platform and processed under the same pipeline, used three relatively large independent datasets, and analyzed both categorical and continuous data. Such remedies may not adequately address the following confounding effects. A non-functional isoform of *ACE2* (dACE2) has recently been discovered to be upregulated in the lung, gastrointestinal, and urinary tract in response to interferon activity and viral infections ^35^. dACE2 might be absent or weakly expressed in normal tissues, but could be induced by the inflammatory tissue microenvironment. Although the survey of TCGA tumors confirmed that *ACE2*, not dACE2, was most expressed in kidney tumors ^35^, it remains possible the expression of dACE2 in various tissues may confound the analysis. The analysis could be also confounded by the disease state. The annotation of disease states for all the samples is cumbersome, if not impossible, given the urgency of this project. Third, when studying cell composition of bulk COVID-19 patient samples, although the computational tool Xcell we employed has been widely used, the exact cell fractions could be quantified more precisely using single-cell technologies. Furthermore, because of heterogeneous cell compositions in the samples, the DE genes might primarily reflect the difference of various cell types rather than that within infected cells. Lastly, as more and more COVID-19 patient clinical data are accumulated, it would be of great interest to further associate creatinine levels with more comorbidities as well as assess efficacy of steroid sex hormones based on real-world evidence. Nevertheless, our analysis of extensive genomic, and clinical data quantified sex differences before and after virus infection.

## Methods

### Data collection

Treehouse OCTAD (T): We downloaded the processed TPM data and phenotype data from the Treehouse project ^21^. We used the same pipeline TOIL to process additional samples and integrated them into a single dataset OCTAD ^36^. This dataset has been used for drug repositioning. In this work, we took the subset of OCTAD where samples have tissue, sex, and age information. The subset includes samples from healthy normal, primary cancer, and adjacent normal tissue samples. This dataset has complete information of sex and age, as well as a fairly complete annotation of tissues. In Figure 1, we only analyzed healthy normal (GTEx) and adjacent normal samples (TCGA). Although healthy normal samples include some diseased patients and some samples are mixed with cancer cells, it remains the best resource for normal tissue samples.

ARCHS4 GPL11154 (A): We downloaded transcript TPM data from the ARCHS4 project which harmonized RNA-Seq sequence data from HiSeq 2000, HiSeq 2500, and NextSeq 500 platforms for human experiments from GEO and SRA. Reads were aligned with Kallisto using a custom cloud computing platform. Human samples were aligned against the GRCh38 human reference genome. The integrated expressions allowed comparing gene expression across tissues in ARCHS4. The metainformation of these samples was downloaded from ARCHS4, and regular expression pattern matching was employed to assist in tissue and age labeling. Only high confidence predictions of tissue and age were considered as labeled data for building machine learning models. We observed a large variation of expression among the three platforms; to facilitate subsequent analysis, we decided to choose HiSeq 200, which is the earliest and most commonly used platform. We drew similar conclusions from the other two platforms, which were not included in the analysis.

GEO GPL570 (G): GPL570 is the most popular microarray platform. CEL files from the GPL570 platform were downloaded using GEOquery. A total of 165 corrupted CEL files were removed from the analysis. Due to size and computational resources, GPL570 was divided into 42 batches (Average: 3477 CELs per batch). Each batch was then normalized with the Affy package using RMA. Selected batches were normalized with justRMA to maintain large batch size. Median was used to merge expression of multiple probes. We included all the samples profiled under this platform. The dataset covers human tissue sample, cultured human cell samples, and those treated with various perturbagens. In order to perform unbiased analysis, we did not exclude cell line samples frequently used at the bench. Sample metadata such as title, source name, and characteristics were pulled out from GEOmetadb.

### Meta-data curation and imputation

For Treehouse OCTAD (T), since sex and age information were relatively complete, we used only labeled data to perform the analysis, which also served as a reference for other datasets. For ARCHS4 GPL11154 (A) and GEO GPL570 (G), only less than 1/3 samples were labeled (Table S1). For samples taken from sex-specific tissues such as prostate and testis, we manually imputed their sex as male. However, the missing rate was still high after this process. Therefore, we further utilized state-of-the-art machine learning models to impute missing labels. Given a dataset, for each target of interest, i.e., sex, age, or tissue, we extracted the gene expression of target-specific (enriched) genes ^37,38^ for all the samples and used labeled data to build a prediction model. For sex and tissue, we built a multi-task deep neural network ^39^ (Figure S6) to predict them together since sex and some tissues are dependent on each other (e.g., only males have prostate/testis samples while breast samples are more likely from females; building models independently for sex and tissue may result in futile predictions such as female-testis). For age prediction, when adding age prediction into a multi-task learning framework, sex and tissue tasks were dominant over age prediction which yielded unsatisfactory results. Therefore, we treated age prediction as a single task and used the extreme gradient boosting (XGBoost) ^40^ to build the model. All the hyper-parameters were tuned using 5-fold cross-validation and the detailed experiment settings can be found in Supplementary Text. After the classifiers were built, we applied it to unlabeled data and predicted their labels. Only predictions with high confidence were kept for later use. For sex, we considered prediction with probability of <0.1 (female) and >0.9 (male) as confident. For multi-class targets (age: 3 classes; tissue: 14 classes), we considered class probability > 0.6 as confident. After that, human inspections were also involved to correct the potentially mislabeled samples based on the characteristics of each sample from the raw source file. Age prediction using gene expression data remains challenging, especially with the context of disease states, so we only predicted three age groups (0–19, 20–59, and >60). We observed that ages 40s and 50s are more likely to be misclassified. In the analysis, we only chose highly confident predictions and focused on the comparison of the younger and older group. In summary, we explored multiple options to ensure that each step produces optimal results. The complete labeled percentage for sex, age, and tissue before and after imputation can be found in Table S1. The model accuracy for all the tasks across all datasets can be found in Table S2, and the visualizations for learned representations are shown in Figure S7.

### Clinical data analysis

Using the Mount Sinai Data Warehouse, we compiled de-identified electronic medical records (EMR) data including age, sex, race, and creatinine levels for inpatients and outpatients confirmed with COVID-19 at Mount Sinai Health System up to 05/10/2020. We normalized the creatinine levels by using x/1.2 for males and x/1.1 for females (x was the creatinine level of the corresponding patient) since the reference range for males is 0.6~1.2 mg/dL and for females is 0.5~1.1 mg/dL. For logistic regression, we set the normalized creatinine > 1 as 1 for both in- and outpatients. In logistic regression, we set sex-female and race-white as the reference. Patient summary statistics can be found in Extended Data and more characteristics of the patients were reported elsewhere ^41,42^

### Statistics and data analysis

For the comparison of sex differences in the proportion of samples with high expression of virus entry proteins, we employed logistic regression with the adjustment of age, tissue, and data source whenever possible. Since smoking status is emerging as an inconclusive factor accounting for sex differences in COVID-19, we adjusted smoking status in a small set (non- smoker: 859, smoker: 1,542), and found men still have higher *ACE2* expression than women (data not shown). Because of the limited size, we did not include smoking status in the main analysis. In tissue analysis, we chose 14 main tissues based on their sample counts and their importance; unknown refers to samples either belonging to other tissues or with low-confidence tissue prediction.

The expression of virus entry proteins is not normally distributed across samples: a majority of them with undetectable expression (TPM < 0.5), and a considerable number of samples with high expression of these entry proteins. We chose the top 10% most highly expressed samples as the high group (label 1) and the remaining as the normal group (label 0). We also explored the thresholds 75%, 80%, 85%, and 95% (the TPM is less than 0.5 at the threshold of 75%). There are variations of ORs among these thresholds, but the conclusions did not change (Figure S8). Such grouping enabled the incorporation of three datasets, the inclusion of samples with undetectable expression, and the mitigation of batch effect between studies. In addition, we applied a similar analysis to individual studies (i.e., GEO GSEs) (Supplementary Text).

The sex difference in continuous gene expression of target genes was evaluated by two metrics: the ratio of average gene expression between female and male groups and the difference of median gene expression between female and male groups. The 95% CI of ratio estimate between female and male groups is obtained by bootstrapping: sampling with replacement and calculating the ratio of average gene expression between female and male for 1000 times, with 2.5% and 97.5% quantile recorded. The difference of median gene expression between women and men is computed using a two-sided Wilcoxon rank-sum test with 95% CI and p-value reported. Since low abundance genes in RNA-Seq samples (TPM < 0.5) are often noisy, these samples were removed accordingly.

### COVID-19 patient sample processing

We searched GEO and SRA using keywords “COVID” and “SARS-CoV-2” and chose those datasets with >3 samples in each sex group (e.g., male healthy, male COVID). We ended with four datasets in the following analysis (Table S4). Two datasets were from upper airways, one dataset was from PMBC and one was from leucocytes. The dataset from leucocytes includes patient severity (ICU vs. non-ICU). Reprocessed raw sequence data available at SRA were downloaded and mapped to the human Hg38 transcriptome using the ENSEMBL GRCh38.p3 annotation using STAR aligner (https://github.com/alexdobin/STAR). The read count mapped on transcriptome was used for DE analysis. All possible comparisons between male and female samples (female-CT vs. male-CT, female-SARS-Cov2 vs. male-SARS-cov2, female-CT vs. female-SARS-Cov2, and male-CT vs. male-SARS-Cov20) were performed. The absolute log2foldchange >=1 with a false discovery rate < 0.01 computed from the EdgeR ^43^ package was chosen to identify DE genes. The DE genes of these comparisons were further compared via vennDiagram R package to find out common genes and specific genes for each comparison. Furthermore, DE genes specific to Control vs. SARS-cov2 in male and Control vs. SARS-cov2 in female were applied to identify enriched pathways through the enrichR API ^44^. The sequence alignment, DE computation, and pathway enrichment were implemented in the OCTAD package ^36^. XCell was employed to infer cell composition ^29^.

## Figures and Tables

**Table 1.** Odds ratios of sex in the prediction of the high expression group in the Healthy dataset and in the Merged dataset. In the regression model, Y is the binary expression level of an entry protein, and X is sex with female being the reference. The all age group is adjusted for age, tissue, and data source, and the older group (>60 years) is adjusted for tissue and data source.

	Samples in the Healthy dataset		Samples in the Merged dataset	
	all ages (n=8,066)	>60 (n=2,849)	all ages (n=220,835)	>60 (n=37,911)
ACE2	1.06 [0.86–1.32]	1.02 [0.72–1.44]	1.25 [1.19–1.30]*	1.15 [1.07–1.23]*
TMPRSS2	1.03 [0.85–1.27]	0.91 [0.65–1.29]	1.28 [1.23–1.34]*	1.32 [1.24–1.42]*
ACE2 & TMPRSS2	0.80 [0.52–1.24]	0.55 [0.27–1.10]	1.16 [1.09–1.24]*	1.12 [1.03–1.22]#

*: p < 0.0001 and

#: 0.009.

## Data Availability

Source data and code is available at GitHub (https://github.com/Bin-Chen-Lab/covid19_sex).
