# Supplementary material for: Sex differences in viral entry protein expression, host responses to SARS-CoV-2, and in vitro responses to sex steroid hormone treatment in COVID-19
Source: Res Sq. 2020 Nov 4:rs.3.rs-100914. Preprint. [Version 1] doi: 10.21203/rs.3.rs-100914/v1 (PMC7654875; doi:10.21203/rs.3.rs-100914/v1)
Supplement: Supplementary file 1 [file 299325db050d7b4a4050c518.DOCX]

**Supplementary** 

**Expression difference of entry proteins between sexes within studies**

We took dataset G (GPL570) as an example. 1,112 datasets (GSEs) have at least five female and five male samples. We further grouped samples into ACE2 high (top 10%) and ACE2 normal as we did in the analysis in the Main text. Even without adjusting for age and tissue, only 180 datasets could build a logistic regression model (with enough samples for each sex and ACE2 group). Without performing the multiple hypothesis testing correction, only 19 datasets had significant sex differences for *ACE2* expression. Therefore, studying sex differences within each study was not feasible.  

**Deep multi-task neural network for sex and tissue prediction**

The architecture of the model predicting sex and tissue is shown in Figure S7.  The gene expressions of sex, age, and tissue enriched genes were used as input features (d1=135), followed by two shared layers (d2=64, d3=32). The joint representations were then fed into two separate network branches (each has dimension d4=32) for sex (2 classes) and tissue (14 classes) prediction, respectively. We used ReLU ^1^ as the activation function for each layer and applied dropout on the shared layers for learning robust shared representations.  Adam ^2^ was used as the optimizer with a learning rate of 0.001. Due to sex and tissue missing at different locations, and in order to fully utilize the available label information, we used samples that have either a label on one or both of the two tasks to train the model. For samples with only one label available (e.g., the sex label available, and the tissue label not available), we applied a mask to the tissue label such that the loss of this sample on tissue task is fixed to zero. The model only updated the sex-specific task parameters and the parameters of the shared layers, and *vice versa* for samples that only had tissue labels. We used 5-fold cross-validation to search for the best dropout rate and re-trained the entire labeled dataset using the best hyperparameter to serve as the final prediction model. All models were implemented using Pytorch ^3^.

**XGBOOST Classifier for age prediction**

Age prediction using gene expression remains challenging, especially with the context of disease states. When adding age prediction into a multi-task learning framework, sex and tissue tasks were dominant over age prediction, which yielded unsatisfactory results. Therefore, we treated age prediction as a single task, and used the extreme gradient boosting (XGBoost) ^4^ to build the model. We used both age and tissue enriched genes as features considering the fact that different tissues usually have different ageing patterns, as preliminary research indicated that the aging signatures are very tissue-specific ^5^. The total features used for age prediction are 130 for GEO GPL570 and 133 for ARCHS4 GPL11154. Looking into them, the labeled data for age groups (younger: 0-19, middle: 20-59, older: >60) have a between-class imbalance problem. For example, the size of the older group in A and the size of the younger group in G are much smaller than the other two groups in each dataset. This imbalance ratio might degrade the prediction performance due to the bias in prediction results toward the majority class. To address this issue, we synthesized new examples from the minority class. Here, we chose the synthetic minority oversampling technique (SMOTE) ^6^, a widely used approach at present. By doing so, the synthetic samples are relatively close in the feature space to the existing samples from the minority class. Afterwards, we performed random under-sampling to make the three groups hold similar prediction distributions across different datasets. Note that oversampling and undersampling were only applied to training data. Implemented by python Scikit-learn ^7^, the hyperparameters of the XGBoost classifier were tuned and the best model was derived by RandomizedSearchCV with 5-fold cross-validation. Consequently, the best-found models were applied to impute unlabeled data for further analyses.

**Tables** 

Table S1. Sample size and labeled percentage of sex, age, and tissue in each dataset before and after imputation.

| Dataset | ARCHS4 GPL11154 (A) | GEO GPL570 (G) | Treehouse OCTAD (T)* |
| --- | --- | --- | --- |
| Total # samples | 60,936 | 142,245 | 17,654 |
| Before Imputation |  |  |  |
| Dataset | ARCHS4 GPL11154 (A) | GEO GPL570 (G) | Treehouse OCTAD (T)* |
| # Labeled Sex | 10,680 (17.5%) | 23,793 (16.7%) | 17,654 (100%) |
| # Labeled Age | 6180 (10.1%) | 18,685 (13.1%) | 17,654 (100%) |
| # Labeled Tissue | 19,702 (32.3%) | 55,685 (39.1%) | 9,677 (54.8%) |
| After Imputation and manual correction |  |  |  |
| Dataset | ARCHS4 GPL11154 (A) | GEO GPL570 (G) | Treehouse OCTAD (T)* |
| # Labeled Sex | 55,754 (91.5%) | 135,961 (95.6%) | - |
| # Labeled Age | 49,176 (80.7%) | 103,631 (72.9%) | - |
| # Labeled Tissue | 49,402 (81.1%) | 92,547 (65.1%) | - |

* Treehouse OCTAD (T) serves as a reference dataset therefore no imputation is applied.

Table S2: Expression of *ACE2*/*TMPRSS2* in different tissues. For each tissue and entry protein pairs, logistic regression was used to compute odds ratios and p values. Y is the binary expression of an entry protein, and X is sex with female being the reference. P values were corrected by the ﻿Benjamini & Hochberg method. The all age group was adjusted by age and data source, and the older group was adjusted by data source. *: p < 0.001; NA: failed to build the model due to limited samples.

|  | All ages |  |  | older group |  |  |
| --- | --- | --- | --- | --- | --- | --- |
|  | *ACE2* | *TMPRSS2* | *ACE2 & TMPRSS2* | *ACE2* | *TMPRSS2* | *ACE2 & TMPRSS2* |
| blood | 0.53(0.36-0.79) | 0.52(0.29-0.88) | 0.2(0.06-0.55) | 0.25(0.04-1.2) | NA | NA |
| bone | 0.48(0.22-0.98) | 0.78(0.4-1.53) | 0.13(0.01-0.72) | NA | NA | NA |
| brain | 0.52(0.33-0.81) | 0.59(0.39-0.88) | 0.87(0.3-2.83) | NA | NA | NA |
| colon | 0.98(0.88-1.09) | 1.07(0.96-1.2) | 1(0.91-1.11) | 1.04(0.91-1.2) | 1.09(0.93-1.29) | 1.04(0.91-1.18) |
| heart | 0.67(0.51-0.88) | 0.68(0.03-17.34) | 0.68(0.03-17.34) | 0.69(0.33-1.44) | NA | NA |
| kidney | 1.45(1.26-1.67)* | 1.06(0.87-1.3) | 1.14(0.9-1.45) | 1.14(0.89-1.46) | 0.84(0.67-1.07) | 0.86(0.67-1.12) |
| liver | 1.46(1.25-1.7)* | 1.21(1.01-1.46) | 1.78(1.34-2.39)* | 1.43(1.05-1.95) | 0.95(0.67-1.34) | 1.14(0.73-1.82) |
| lung | 0.9(0.78-1.04) | 0.71(0.64-0.78)* | 0.68(0.56-0.82)* | 0.72(0.58-0.9) | 0.68(0.58-0.79)* | 0.57(0.42-0.76)* |
| pancreas | 0.89(0.71-1.12) | 0.79(0.63-0.99) | 0.71(0.51-0.97) | 0.72(0.5-1.02) | 1.09(0.76-1.56) | 0.73(0.45-1.19) |
| skin | 1.53(1.22-1.92) | 1.33(0.75-2.42) | 0.91(0.35-2.34) | 0.12(0.03-0.36) | NA | NA |
| small intestine | 1.24(0.81-1.92) | 0.7(0.48-1.03) | 0.67(0.45-0.97) | 0.17(0.05-0.47) | 0.18(0.07-0.41) | 0.15(0.06-0.35)* |
| unknown | 1.12(1.05-1.2) | 1.92(1.79-2.06)* | 1.62(1.44-1.81)* | 1.57(1.4-1.76)* | 2.26(2.04-2.52)* | 1.9(1.63-2.22)* |

Table S3. 5-fold average accuracy (standard deviation) for all datasets and targets of interest.

|  | ARCHS4 GPL11154 (A) | GEO GPL570 (G) |
| --- | --- | --- |
| Sex | 0.953 (0.026) | 0.919 (0.044) |
| Age | 0.862 (0.014) | 0.754 (0.028) |
| Tissue | 0.858 (0.038) | 0.887 (0.017) |

Table S4: COVID-19 patient datasets

| **GEO ID** | **Tissue** | **Male** |  | **Female** |  |
| --- | --- | --- | --- | --- | --- |
|  |  | **COVID-19** | **Control** | **COVID-19** | **Control** |
| GSE152075 | Nasal swab | 176 | 24 | 201 | 30 |
| GSE156063 | upper airway (NP/OP swab) | 43 | 45 | 50 | 55 |
| GSE152418 | PBMC | 7 | 8 | 9 | 9 |
| GSE157103 | Leucocytes | 62 | 12 | 38 | 13 |

**Figures** 

Figure S1. Data collection and analysis workflow. We defined high-expression as the top 10% within individual datasets (T, G, A) and explored other thresholds as well. ARCHS4: All RNA-seq and CHIP-Seq Signature Search Space developed by Ma'ayan Laboratory; GEO: Gene Expression Omnibus; GTEx: The Genotype-Tissue Expression; Kallisto: program for quantifying abundances of transcripts from bulk and single-cell RNA-Seq data; Meta500: 500 metastatic cancers; RMA: Robust Multi-array Average; SRA: Sequence Read Archive; TARGET: The Therapeutically Applicable Research to Generate Effective Treatments; TCGA: The Cancer Genome Atlas; TOIL: A RNASeq processing pipeline developed by UC Santa Cruz; Treehouse: Treehouse Childhood Cancer Initiative; TPM: Transcripts Per Million.

Figure S2. *TMPRSS2* expression across (A) age groups and (B) across tissues in three datasets Treehouse (T), ARCHS4 (A), and GPL570 (G). In T, only normal and peritumoral tissue samples were selected. Due to the limited sample size (n), in the 0-19 age group, and blood and bone tissues were not included in (A) and (B), respectively.  For T and A, samples with TPM < 0.5 were removed. M/F represents the ratio of mean expression between male and female. The 95% confidence interval of ratio was obtained using bootstrapping, i.e., sampling with replacement and calculating the ratio for 1000 times with 2.5% and 97.5% quantile reported. P value was computed from a two-sided Wilcoxon rank sum test on the difference of median expression between male and female group.  * means P < 0.001.The bar and the dashed line show the mean of expressions in each group and the mean of all expressions, respectively.

Figure S3. Expression of *ACE2* in diseases with very high expression of *ACE2*. We first identified diseases with very high expression of *ACE2* and then searched GEO for related datasets with both disease and control samples.  Control samples are often matched normal tissues. Expression values were downloaded from GEO and t-test was performed to compute the significance. Only significant pairs are listed here (p < 0.05). Due to the limited sample size, we did not examine sex differences for those samples.

Figure S4. Correlation between ACE2 and steroid hormone receptors. (A) Expression correlation between ACE2 and sex steroid hormone receptors in kidney. All kidney samples in GPL570 were used. Similar correlations were observed in men and women, separately (data not shown). (B) Association between transcription factor target genes and ACE2 co-expressed genes. The target genes of ESR1 and AR were downloaded from enrichR ^8^ (ENCODE_and_ChEA_Consensus_TFs_from_ChIP-X). Transcription factor regulations listed in ENCODE and ChEA were inferred from genome-wide experiments such as ChIP-chip and ChIP-Seq. ESR2 target genes were not provided in this datasets. Top 200 positively/negatively co-expressed genes with ACE2 were computed using GTEx data. Only the tissue with over 30 samples was selected. A hypergeometric test was used to compute the significance. The heatmap was colored based on the -log(10) of p values.  

Figure S5: Odds ratio (OR) and enrichment of cells in COVID-19 patients. Odds ratios of different cells between ICU and non-ICU patients (A) in male and (B) in female. Logistic regression was performed with non-ICU patients as the reference and age adjusted. P values were corrected by a multiple hypothesis test and cell types with an adjusted p value <= 0.05 were selected. Heatmaps show the enrichment value of cells in male (c) and (d) in female. 

Figure S6: Dose‐response curve (DRC) of hormone drugs in (A) Vero and (B) Calu-3. The red line indicates cell viability, and the blue line indicates inhibition of SARS‐CoV‐2 infection. SARS‐CoV‐2 infectivity was measured by immunofluorescence of SARS‐CoV‐2 N protein. Each point is a mean of duplicate experiments ± standard deviation. IC50, CC50, and SI are noted below each graph. CC50, half maximal cytotoxic concentration; IC50, half maximal inhibitory concentration; SARS‐CoV‐2, severe acute respiratory syndrome coronavirus 2; SI, selective index.

Figure S7. Deep multi-task neural network for sex and tissue prediction. The architecture consists of three shared layers followed by two separate branches for each of the tasks. The numbers represent the dimension of each layer. 

Figure S8. T-SNE plot of learned representations (d=32) of the validation set for tissue prediction. Different colors represent different tissue types.

Figure S9: Data related to Table 1. Multiple thresholds were used to define high expression groups. The triangle means a significant odds ratio.

**References**

1.	Nair, V. & Hinton, G. E. Rectified linear units improve restricted boltzmann machines. in *Proceedings of the 27th International Conference on International Conference on Machine Learning* 807–814 (Omnipress, 2010).

2.	Kingma, D. P. & Ba, J. Adam: A Method for Stochastic Optimization. *ArXiv14126980 Cs* (2017).

3.	Paszke, A. *et al.* PyTorch: An Imperative Style, High-Performance Deep Learning Library. in *Advances in Neural Information Processing Systems 32* (eds. Wallach, H. et al.) 8026–8037 (Curran Associates, Inc., 2019).

4.	Chen, T. & Guestrin, C. XGBoost: A Scalable Tree Boosting System. *Proc. 22nd ACM SIGKDD Int. Conf. Knowl. Discov. Data Min.* 785–794 (2016) doi:10.1145/2939672.2939785.

5.	Yang, J. *et al.* Synchronized age-related gene expression changes across multiple tissues in human and the link to complex diseases. *Sci. Rep.* **5**, 15145 (2015).

6.	Chawla, N. V., Bowyer, K. W., Hall, L. O. & Kegelmeyer, W. P. SMOTE: Synthetic Minority Over-sampling Technique. *J. Artif. Intell. Res.* **16**, 321–357 (2002).

7.	Pedregosa, F. *et al.* Scikit-learn: Machine Learning in Python. *J. Mach. Learn. Res.* **12**, 2825–2830 (2011).

8.	Chen, E. Y. *et al.* Enrichr: interactive and collaborative HTML5 gene list enrichment analysis tool. *BMC Bioinformatics* **14**, 128 (2013).
